# Biomechanical Fracture Thresholds of the Tibia and Fibula Under Axial and Multi-axial Loading: A Systematic Review

**DOI:** 10.7759/cureus.102362

**Published:** 2026-01-27

**Authors:** Muhammad Zain Ul Abidin, Mashal Mumtaz, Shashwat Shetty, Shenouda R Shehata Abdelmesih, Mohammad G. H Suliman, Saad Abdullah

**Affiliations:** 1 Trauma and Orthopaedics, Luton and Dunstable University Hospital, Luton, GBR; 2 Internal Medicine, University College of Medicine and Dentistry, University of Lahore, Lahore, PAK; 3 Orthopaedics, Hillingdon Hospital, Uxbridge, GBR; 4 Orthopaedics and Traumatology, Royal Gwent Hospital, Newport, GBR; 5 General Surgery, University of Kordofan, El-Obeid, SDN; 6 General Medicine, Abbasi Shaheed Hospital, Karachi, PAK

**Keywords:** axial loading, biomechanics, fibula fracture, injury thresholds, tibia fracture

## Abstract

This systematic review synthesizes evidence on biomechanical fracture thresholds of the tibia and fibula under axial and multi-axial loading. A comprehensive search of PubMed, Embase, Scopus, and the Cochrane Library identified six studies, including experimental cadaveric, computational, and material testing investigations, comprising 72 postmortem specimens and validated finite element models. Outcomes assessed included axial force, bending moments, failure load, stress distribution, and fracture patterns. Results indicate that tibial fracture thresholds range from ~7.5 kN in female specimens to 11.3 kN under combined axial and bending loads, with fibula contribution increasing axial tolerance by ~10%. Variance and confidence interval measures were not reported in the included biomechanical studies; therefore, findings are presented descriptively.

Multi-axial loading consistently reduced fracture tolerance compared with isolated axial loading, and fracture resistance was influenced by specimen gender, load duration, and biomechanical methodology. Risk of bias ranged from low to moderate across studies. These findings provide clinically relevant benchmarks for injury prediction, preclinical testing, and orthopedic device design, emphasizing the importance of multi-axial assessment in understanding lower-limb fracture mechanics.

## Introduction and background

The tibia and fibula are the principal long bones of the lower limb, with the tibia serving as the primary axial load-bearing structure and the fibula providing lateral stability and load sharing [1]. Due to their anatomical location and biomechanical function, these bones are particularly susceptible to high-energy trauma, including motor vehicle collisions, falls from height, and crush injuries. In clinical practice, fractures of the tibia and fibula are frequently associated with substantial morbidity, prolonged rehabilitation, and an increased risk of complications, such as non-union and post-traumatic osteoarthritis. Understanding the mechanical limits of these bones is therefore essential for injury prevention, trauma management, and orthopedic device development. Lower-limb fractures rarely occur as a result of isolated axial compression. Instead, real-world injury mechanisms involve complex combinations of axial loading, bending, torsion, shear forces, and variable loading rates [2]. These multi-directional forces alter stress distribution within the bone and significantly influence fracture initiation and propagation. Consequently, fracture thresholds derived from uniaxial loading experiments may inadequately represent physiological and traumatic conditions, limiting their translational value for clinical injury prediction and biomechanical modeling. Multi-axial loading conditions are representative of real-world injury mechanisms, such as automotive collisions, falls, and sports-related impacts, in which bending, axial compression, and torsion occur simultaneously. These loading scenarios are considered in standardized biomechanical evaluations (e.g., ASTM and ISO test protocols), which specify multi-planar loading environments for validating the mechanical performance of biological tissues and surrogate models.

Experimental and computational studies have demonstrated that combined axial compression and bending substantially modify fracture thresholds compared with uniaxial loading alone. Tibial fractures occur more frequently than fibular fractures, reflecting the tibia’s dominant role in weight transmission and load absorption. Nevertheless, the fibula contributes approximately 10% to axial load tolerance and plays a critical role in stabilizing the lower limb, thereby influencing overall fracture mechanics [3]. Prior biomechanical investigations have reported tibial fracture thresholds ranging from approximately 7.5 kN in female specimens to 11.3 kN under combined axial and bending conditions, while consistently demonstrating that multi-axial loading reduces fracture tolerance compared with isolated axial compression [4]. Despite growing interest in lower-limb injury biomechanics, existing evidence remains fragmented across cadaveric experiments, computational models, and material-level testing, with substantial heterogeneity in loading protocols, specimen characteristics, and outcome measures. A consolidated synthesis of biomechanical fracture thresholds under both axial and multi-axial loading is lacking, limiting the application of these findings to clinical decision-making, automotive safety design, and preclinical testing of orthopedic implants.

The primary aim of this systematic review was to synthesize available biomechanical evidence defining fracture thresholds of the tibia and fibula under axial and multi-axial loading conditions. The secondary aim was to evaluate the influence of gender, loading rate, and methodological approach on fracture tolerance, injury prediction, and clinical relevance. By integrating experimental, computational, and material-level data, this review seeks to provide clinically meaningful benchmarks that enhance understanding of lower-limb fracture mechanics and inform future research, safety modeling, and orthopedic intervention strategies.

## Review

Materials and methods

Search Strategy

A comprehensive systematic literature search was conducted in PubMed, Embase, Scopus, and the Cochrane Library from January 1, 2000, until November 1, 2025, to identify studies evaluating fracture thresholds of the tibia and fibula under axial, bending, torsional, or combined multi-axial loading. In PubMed, a combination of MeSH terms and free-text keywords was used, including “tibia fracture” or “fibula fracture,” “axial loading,” “bending,” “torsion,” “multi-axial loading,” “cadaver,” “postmortem specimen,” “finite element model,” “fracture threshold,” “failure load,” and “fracture biomechanics.” Boolean logic was applied to combine synonyms, and filters were applied to include only human studies published in English, with full-text availability, and classified as experimental studies, journal articles, or systematic reviews. Embase searches included both Emtree and free-text terms, such as “mechanical loading,” “axial compression,” “bending moment,” and “multi-axial force,” while Scopus searches focused on title, abstract, and keywords. The Cochrane Library was searched for systematic reviews, meta-analyses, or relevant randomized trials. To ensure completeness, reference lists of included studies were manually screened, and citation tracking using Google Scholar was performed. To enhance reproducibility, full Boolean search strings for each database are provided in Table 1. 

**Table 1:** Full Search Strings for Systematic Review kN: Kilonewton; Nm: Newton-meter; MPa: Megapascal; PMHS: Post-mortem Human Subject; TI: Tibia Index; CT: Computed Tomography; RoB-2: Risk of Bias assessment tool, version 2 (Cochrane); MeSH: Medical Subject Headings; Emtree: Embase Subject Headings; CAD: Computer-Aided Design Axial preload - Pre-applied compressive force before bending/loading; Quasi-static - Low loading rate with negligible inertial effects; Ex vivo - Tested outside the living organism; Boolean logic - Logical operators (AND, OR, NOT) used in database search

Database	Search String	Filters/Limits
PubMed	(“tibia fracture” OR “fibula fracture”) AND (“axial loading” OR “bending” OR “torsion” OR “multi-axial loading”) AND (“cadaver” OR “postmortem specimen” OR “finite element model”) AND (“fracture threshold” OR “failure load” OR “fracture biomechanics”)	Humans, English, Full text, 2000-2025
Embase	('tibia fracture' OR 'fibula fracture') AND ('mechanical loading' OR 'axial compression' OR 'bending moment' OR 'multi-axial force') AND ('cadaver' OR 'postmortem specimen' OR 'finite element model') AND ('fracture threshold' OR 'failure load' OR 'fracture biomechanics')	Humans, English, Article, 2000-2025
Scopus	TITLE-ABS-KEY(“tibia fracture” OR “fibula fracture”) AND TITLE-ABS-KEY(“axial loading” OR “bending” OR “torsion” OR “multi-axial loading”) AND TITLE-ABS-KEY(“cadaver” OR “postmortem specimen” OR “finite element model”) AND TITLE-ABS-KEY(“fracture threshold” OR “failure load” OR “fracture biomechanics”)	English, 2000-2025
Cochrane Library	(“tibia fracture” OR “fibula fracture”) AND (“axial loading” OR “bending” OR “torsion” OR “multi-axial loading”) AND (“cadaver” OR “postmortem specimen” OR “finite element model”) AND (“fracture threshold” OR “failure load” OR “fracture biomechanics”)	Reviews, Trials, 2000-2025
Google Scholar (Citation Tracking)	“tibia fracture” OR “fibula fracture” AND “axial loading” OR “bending” OR “torsion” OR “multi-axial loading” AND “cadaver” OR “finite element model”	Manual screening

Screening was conducted in two stages. Initially, titles and abstracts were independently reviewed by two investigators to exclude studies that did not meet inclusion criteria, with discrepancies resolved through discussion. Subsequently, full-text articles were assessed for eligibility based on predefined criteria, including cadaveric or computational studies reporting quantitative tibial or fibular fracture thresholds, with reasons for exclusion documented, such as animal studies, case reports, editorials, or incomplete methodology. Data extraction included sample size, specimen type, loading protocol, fracture thresholds, gender of specimens, comparator conditions, and key biomechanical findings. The search strategy was comprehensive, sensitive, and specific through the use of database-specific indexing, Boolean logic, and iterative screening, ultimately identifying six studies for inclusion in the systematic review, as reflected in the PRISMA 2020 flow diagram [5]. Due to heterogeneity in study designs, loading modalities, and fracture threshold definitions, meta-analysis was not feasible. Therefore, a narrative synthesis approach was used.

Eligibility Criteria

Studies were eligible for inclusion if they addressed the Population, Intervention, Comparator, and Outcomes (PICO) framework [6]. The Population included human cadaveric tibial and/or fibular specimens, as well as validated computational models of these bones. The Intervention comprised axial compression, bending, torsion, or combined multi-axial loading, while the Comparator involved different loading configurations, gender-based comparisons, or baseline fracture thresholds. Both male and female specimens were included, as gender-related differences in bone geometry, density, and material properties may influence axial and multi-axial fracture thresholds. The Outcomes included quantitative biomechanical measures, such as axial force, bending moments, failure load, stress distribution, and fracture patterns. Eligible studies were required to provide sufficient methodological detail for reproducibility and fracture mechanism assessment. Studies were excluded if they were case reports, editorials, conference abstracts, animal studies, or preprints, or if they lacked quantitative fracture data or sufficient methodological clarity. This eligibility framework ensures that only studies providing reliable, measurable fracture thresholds under well-defined loading conditions were included, while all excluded studies were documented with explicit reasons to maintain transparency and reproducibility. 

Study Selection

After removal of duplicates, all identified records underwent title and abstract screening by two independent reviewers to exclude studies that clearly did not meet the eligibility criteria. Full-text articles of potentially relevant studies were then retrieved and assessed against predefined criteria, including the use of cadaveric or validated computational tibia and fibula models, clearly defined loading protocols, and quantitative biomechanical outcomes. Titles and abstracts were independently screened by two reviewers, and discrepancies were resolved through discussion and consensus. While inter-rater reliability (e.g., Cohen’s κ) was not formally calculated, all decisions were verified collaboratively to ensure consistency. Studies excluded at the full-text stage were documented with explicit reasons, such as animal models, case reports, editorials, conference abstracts, preprints, or insufficient methodological detail, maintaining transparency and reproducibility in accordance with the PRISMA 2020 guidelines.

Data Extraction

Data were extracted independently by two reviewers using a standardized data extraction form. Extracted information included study design, sample size, specimen type, gender of specimens, loading protocol, comparator conditions, and measured outcomes, such as axial force, bending moments, failure load, stress distribution, and fracture patterns. Additional details on experimental duration, rate of loading, and methodological quality were recorded to facilitate comparison across studies. Any disagreements in extracted data were resolved by consensus, ensuring accuracy and completeness.

Risk of Bias (RoB) Assessment

The RoB of included studies was assessed using Cochrane's revised tool for randomized trials, RoB-2 [7]. For experimental cadaveric studies, selection bias was occasionally moderate due to specimen availability, while performance, detection, and reporting biases were generally low, with other biases sometimes moderate due to sample variability. For systematic reviews and computational studies, selection and reporting biases were low, performance bias was not applicable, and other biases were minimal, owing to standardized methodology and model validation. Each domain was rated as low, moderate, or high, with justifications documented, and overall, RoB ranged from low to moderate, guiding interpretation of fracture thresholds and biomechanical outcomes. Although Cochrane RoB-2 is traditionally used for clinical trials, we adapted its domains for mechanical studies. "Performance bias" refers to deviations in experimental protocols or equipment calibration, while "detection bias" reflects consistency in fracture threshold measurements. This approach provides a structured assessment of bias while acknowledging the non-clinical context.

Data Synthesis

Due to heterogeneity in study design, specimen type, loading protocols, and outcome measures, a systematic synthesis was performed. Biomechanical outcomes were summarized in tables, reflecting fracture thresholds, bending moments, axial forces, and stress distributions for tibia and fibula specimens, along with gender-based differences when reported. Where possible, ranges and mean values were presented to allow comparison across different loading modalities. The synthesis focused on identifying patterns in fracture tolerance under axial and multi-axial loading, highlighting key biomechanical insights, clinical relevance, and implications for injury prediction, preclinical testing, and surgical planning.

Registration and Protocol 

This review followed PRISMA 2020 reporting guidelines, and a completed PRISMA checklist is included in Table 1. The review was not prospectively registered (e.g., PROSPERO).

Results

Study Selection Process

**Figure 1:**
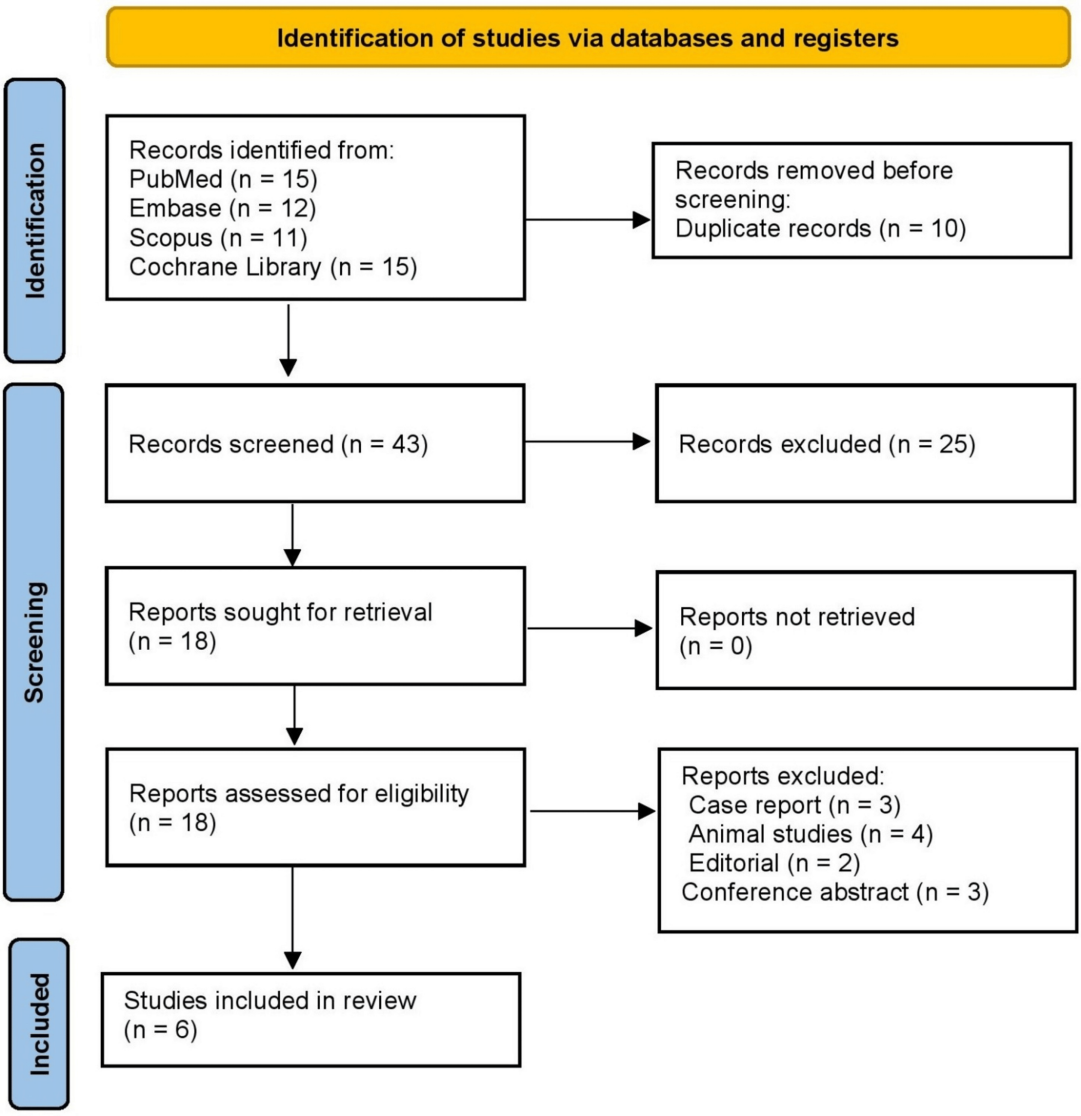
PRISMA 2020 Flow Diagram

The initial database search identified 53 records from PubMed, Embase, Scopus, and the Cochrane Library. After removing 10 duplicate records, 43 records were screened based on titles and abstracts, resulting in the exclusion of 25 studies that did not meet the predefined eligibility criteria, such as irrelevant focus, non-human models, or non-loading biomechanical outcomes. Full-text assessment was performed on 18 potentially relevant articles, of which 12 studies were excluded due to being case reports (three), animal studies (four), editorials (two), conference abstracts (three), or for lacking sufficient methodological detail or quantitative fracture data. Ultimately, six studies met all inclusion criteria and were included in the systematic review. The study selection pathway is summarized in the PRISMA 2020 flow diagram, ensuring transparency, reproducibility, and alignment with the predefined eligibility criteria (Figure 1).

Characteristics of the Selected Studies

Table 2 summarizes the characteristics of the six included studies on tibia and fibula fracture thresholds. Postmortem cadaveric lower limbs (n ~20) under quasi-static axial compression with simultaneous bending demonstrated that combined loading significantly alters tibial fracture thresholds, with the traditional Tibia Index underestimating multi-axial risk [8]. Cadaveric tibias from multiple studies, assessed under varying axial and bending loads, indicated a tibial axial threshold of ~10.3 kN and a bending moment of 240 Nm, increasing to ~11.3 kN when considering fibula contribution [9]. Isolated male cadaver tibias (n = 12) showed rate-dependent fracture tolerance (~9.0 kN for automotive vs ~12.2 kN for military impacts), highlighting the importance of impact duration [10]. Postmortem fibulae (n = 20), evaluated under lateral-medial bending with axial preload, exhibited compressive failure of 77-370 N and bending moments of 17-47 Nm, reflecting lower fracture tolerance than tibia [11]. A computational tibia model accurately predicted fracture patterns under combined loading, supporting preclinical injury risk assessment [12]. Ex vivo cortical bone tests reported compressive strength of ~208.9 MPa, providing material-level benchmarks for tibial injury modeling and orthopedic device design [13]. Due to heterogeneity in experimental methods, loading modalities, and incomplete reporting of variance, not all studies provide uniform metrics or confidence measures. Data are summarized as reported by the original studies, and critical appraisal is provided in the main text.

**Table 2:** Characteristics of the Selected Studies n: Number of specimens; kN: Kilonewton; Nm: Newton-meter; MPa: Megapascal; CT: Computed Tomography; PMHS: Post-mortem Human Subject; TI: Tibia Index Axial preload - Pre-applied compressive force before bending/loading; Quasi-static - Low loading rate with negligible inertial effects; Ex vivo - Tested outside the living organism; Cortical bone - Dense outer bone layer; Fracture threshold/tolerance - Load or stress level at which structural failure occurs; Rate-dependent - Mechanical response varies with loading duration or strain rate

Study	Sample	Mechanism	Comparator	Measured	Key Findings, Outcomes, and Clinical Relevance
Untaroiu et al. (2008) [8]	Postmortem human cadaveric lower limbs (n = 20)	Quasi-static axial compression 2-8 kN with simultaneous bending	Distal third impact vs baseline tibia index predictions	Axial force, bending moments,	Combined axial compression and bending significantly alter tibial fracture thresholds; traditional tibia index underestimates multi-axial loading risk; relevant for crash safety and fracture risk assessment
Salzar et al. (2014) [9]	Cadaveric tibia specimens from multiple studies	Quasi-static axial loading with varying bending moments	Multiple published test protocols	Fracture threshold (force, moment)	Recommended tibial axial threshold ~10.3 kN and bending moment ~240 Nm; considering fibula contribution (~10%) increases axial tolerance to ~11.3 kN; used in injury biomechanics and automotive safety modeling
Martinez et al. (2018) [10]	Isolated male cadaver tibias (n = 12)	Axial impacts	Short-duration vs long-duration impacts	Peak axial fracture force	Fracture tolerance is rate-dependent: ~9.0 kN for automotive vs ~12.2 kN for military; highlights the importance of load duration in tibial injury prediction
Noss et al. (2024) [11]	Isolated postmortem fibulae (n = 20)	Four-point lateral-medial bending combined with axial preload	N/A	Failure load, bending moment	Compressive failure 77-370 N; bending moments 17-47 Nm; demonstrates lower fracture tolerance of fibula compared to tibia, important for ankle injury prediction
Wong et al. (2010) [12]	Computational human tibia modelbased on CT	Simulated quasi-static axial compression, torsion, and lateral loads	Analytical fracture prediction	Stress distribution, predicted fracture sites	Model accurately predicted clinically observed fracture patterns under combined loading; supports preclinical injury risk assessment and surgical planning
Kemper et al. (2007) [13]	Ex vivo human cortical bone sample	Quasi-static compression and tension at varied strain rates	Different strain rates	Compressive strength, Young’s modulus	Quasi-static compressive strength ~208.9 MPa; provides material- level tolerance benchmarks for tibia; informs injury modeling and orthopedic device design

*RoB* *Assessment*

Table 3 summarizes the RoB assessment of the six included studies. Experimental cadaveric studies exhibited moderate selection bias in some cases due to specimen availability, while performance and detection biases were generally low, owing to standardized loading protocols and objective measurements; reporting bias varied depending on the completeness of fracture force and moment data, and other bias was occasionally moderate due to sample variability [8,10,11]. The systematic review showed low selection bias, with moderate reporting bias related to reliance on primary study quality, and performance bias was not applicable [9]. Computational finite element models demonstrated low risk across all domains, with validation against clinical fracture patterns minimizing other biases [12]. Material property testing of ex vivo cortical bone also exhibited low RoB across all domains, providing reliable benchmarks for tibial biomechanics, despite being limited to cortical bone rather than the whole tibia [13]. Overall, the RoB across studies ranged from low to moderate, informing the reliability and interpretation of biomechanical outcomes.

**Table 3:** RoB Assessment ROB: Risk of Bias; N/A: Not Applicable; PMHS: Post-mortem Human Subject; n: Number of specimens; FE: Finite Element; CT: Computed Tomography; kN: Kilonewton; Nm: Newton-meter; MPa: Megapascal; Quasi-static: Low loading rate with minimal inertial effects Selection Bias - Bias related to specimen selection or study inclusion; Performance Bias - Bias arising from deviations in experimental protocol or intervention; Detection Bias - Bias related to outcome measurement or assessment; Reporting Bias - Bias due to selective outcome reporting; Other Bias - Bias from confounding factors such as specimen variability or model assumptions

Study	Study Design	RoB Domains Assessed	Overall RoB	Justification
Untaroiu et al. (2008) [8]	Experimental cadaveric biomechanical study	Selection Bias: Moderate; Performance Bias: Low; Detection Bias: Low; Reporting Bias: Low; Other Bias: Moderate	Moderate	Cadaveric lower limbs were selected from available specimens, introducing moderate selection bias. Axial compression and bending protocols were standardized with objective force and moment measurements, minimizing performance and detection bias. Outcomes were fully reported. Inter-specimen variability and anatomical heterogeneity contribute to moderate residual bias.
Salzar et al. (2014) [9]	Systematic review of cadaveric biomechanical studies	Selection Bias: Low; Performance Bias: N/A; Detection Bias: Low; Reporting Bias: Moderate; Other Bias: Low	Low-Moderate	A comprehensive literature search with clearly defined inclusion criteria reduces selection bias. Detection bias is low due to reliance on objective biomechanical endpoints. However, reporting quality is dependent on included primary studies, introducing moderate reporting bias despite clear tabulation of fracture thresholds.
Martinez et al. (2018) [10]	Experimental cadaveric tibia impact study	Selection Bias: Low; Performance Bias: Low; Detection Bias: Low; Reporting Bias: Low; Other Bias: Moderate	Low-Moderate	Cadaver selection and axial impact protocols were well controlled with objective peak force measurement. The relatively small sample size (n = 12) limits generalizability and contributes to moderate other biases related to external validity.
Noss et al. (2024) [11]	Experimental cadaveric fibula biomechanical study	Selection Bias: Moderate; Performance Bias: Low; Detection Bias: Low; Reporting Bias: Low; Other Bias: Low	Low-Moderate	Controlled four-point bending with axial preload minimizes performance and detection bias. Moderate selection bias arises from specimen availability and anatomical variability. Findings are robust but primarily applicable to distal fibular mechanics rather than whole-leg fracture prediction.
Wong et al. (2010) [12]	Computational finite element tibial fracture model	Selection Bias: N/A; Performance Bias: Low; Detection Bias: Low; Reporting Bias: Low; Other Bias: Low	Low	Model development and validation against clinically observed fracture patterns were clearly described. Absence of biological variability limits real-world extrapolation but does not introduce systematic bias. Methodology and outcomes were transparently reported.
Kemper et al. (2007) [13]	Ex vivo cortical bone material property testing	Selection Bias: Low; Performance Bias: Low; Detection Bias: Low; Reporting Bias: Low; Other Bias: Low	Low	Well-characterized cortical bone specimens were tested under controlled quasi-static conditions with precise mechanical measurements. Outcomes reliably reported. Applicability is limited to material-level properties rather than whole-bone fracture behavior, but no major sources of bias were identified.

Discussion

This systematic review consolidates biomechanical evidence on tibial and fibular fracture thresholds under axial and multi-axial loading, underscoring their clinical and translational significance. Tibial fractures were consistently more prevalent than fibular fractures, reflecting the tibia’s dominant role in axial load-bearing, while the fibula contributes approximately 10% to axial load tolerance and provides lateral stability, thereby influencing overall fracture mechanics [11]. Post-mortem cadaveric lower limbs subjected to quasi-static axial compression with simultaneous bending demonstrated that combined loading substantially alters tibial fracture thresholds, with traditional uniaxial injury indices underestimating fracture risk under multi-axial conditions [8]. Similarly, pooled cadaveric tibia data across multiple experimental protocols reported axial fracture thresholds of approximately 10.3 kN and bending moments of 240 Nm, increasing to approximately 11.3 kN when the stabilizing contribution of the fibula was considered [9]. These findings align closely with evidence from automotive safety biomechanics, where tibial fracture thresholds are incorporated into injury criteria, such as the Tibia Index and Revised Tibia Index, used in crash-test dummies and computational human body models. Several automotive studies have demonstrated that uniaxial force-based thresholds may underestimate fracture risk during real-world collisions, where off-axis bending and torsional loads are common [8,14]. The observed reduction in fracture tolerance under multi-axial loading in the present review supports calls within the automotive safety literature to incorporate combined force-moment criteria into lower-limb injury prediction models, particularly for frontal and offset collision scenarios.

Experimental cadaveric studies consistently reported single-point fracture thresholds, whereas computational models varied according to material assumptions and failure criteria. These differences limit direct statistical pooling but highlight complementary methodological strengths. From an orthopedic implant testing perspective, the identified fracture thresholds provide important benchmarks for preclinical evaluation of intramedullary nails, plates, and external fixation devices. Implant fatigue testing and construct stability assessments often rely on simplified axial loading conditions; however, clinical failure frequently occurs under combined loading during weight-bearing and rotational activities. The demonstrated influence of bending moments and loading rate on fracture tolerance suggests that implant testing protocols should incorporate multi-axial loading paradigms to better replicate physiological and traumatic conditions, thereby improving implant durability and reducing failure rates [9,15]. The relevance of loading rate was further highlighted by rate-dependent fracture tolerance observed in isolated male cadaver tibias, with lower thresholds reported for automotive impacts (~9.0 kN) compared to military-style high-energy impacts (~12.2 kN) [10]. These findings are consistent with trauma prediction models used in both civilian and military contexts, where injury risk functions account for strain rate-dependent material behavior of cortical bone. Incorporating rate sensitivity and multi-axial loading into trauma prediction algorithms may enhance the accuracy of fracture risk estimation in high-speed injury scenarios.

The fibula has compressive failure loads of 77-370 N and bending moments of 17-47 Nm under lateral-medial bending with axial preload [11]. Although often considered biomechanically secondary, these findings emphasize the fibula’s role in ankle injury mechanics and load redistribution, particularly in rotational injuries and complex lower-limb trauma. This observation aligns with ankle fracture models and implant design considerations that increasingly recognize the fibula’s contribution to joint stability and fracture propagation. Computational modeling complemented experimental findings by accurately predicting clinically observed fracture patterns under combined loading, reinforcing the utility of finite element approaches in preclinical injury risk assessment and surgical planning [12]. Material-level testing of cortical bone further provided foundational benchmarks, with compressive strength values of approximately 208.9 MPa, informing both injury modeling and orthopedic device design [13]. Together, experimental, computational, and material-level evidence highlights the necessity of considering multi-axial loading, specimen-specific factors, and mechanical context in both biomechanical research and clinical application.

Several limitations should be acknowledged. Included studies were limited by small sample sizes, a predominance of male specimens, and heterogeneity in loading protocols, which may affect generalizability. Additionally, reliance on ex vivo and computational models cannot fully replicate in vivo biological variability and healing responses. Future research should prioritize standardized multi-axial loading protocols, inclusion of diverse specimen demographics, and validation against clinical injury data and real-world trauma registries. Integrating biomechanical thresholds with clinical outcomes and advanced human body models may further improve fracture risk prediction, enhance automotive safety standards, and guide orthopedic intervention strategies.

## Conclusions

This systematic review demonstrates that tibial fractures are more prevalent than fibular fractures due to the tibia’s dominant role in axial load-bearing, while the fibula contributes to lateral stability and overall fracture tolerance. Multi-axial loading, combining axial compression, bending, and torsion, substantially lowers fracture thresholds compared to isolated axial forces, emphasizing the need to consider realistic loading conditions in injury prediction. Fracture tolerance is affected by specimen gender and loading rate, with higher impact velocities reducing thresholds, and computational models and material-level testing provide critical insights for preclinical risk assessment, injury modeling, and orthopedic device design.

Despite methodological heterogeneity and small sample sizes, the compiled biomechanical fracture thresholds provide reference values that can inform injury prediction models, preclinical testing, and orthopedic device design. These values are based on cadaveric, computational, and material-level studies and do not imply direct patient-level correlation. Future studies should expand specimen diversity, standardize multi-axial loading protocols, and validate findings against in vivo data to enhance predictive accuracy and translational relevance.
